# The impact of impaired sleep quality on symptom change and future exacerbation of chronic obstructive pulmonary disease

**DOI:** 10.1186/s12931-023-02405-6

**Published:** 2023-03-30

**Authors:** Ling Lin, Qing Song, Jiaxi Duan, Cong Liu, Wei Cheng, Aiyuan Zhou, Yating Peng, Zijing Zhou, Yuqin Zeng, Yan Chen, Shan Cai, Ping Chen

**Affiliations:** 1 Department of Respiratory and Critical Care Medicine, Second Xiangya Hospital, Central South University, 139 Renmin Middle Road, Changsha, Hunan 410011 China; 2 Research Unit of Respiratory Disease, Central South University, Changsha, Hunan 410011 China; 3 Diagnosis and Treatment Center of Respiratory Disease, Central South University, Changsha, Hunan 410011 China; 4 Geriatrics Department, Xiangya Hospital, Central South University, Changsha, Hunan China; 5 Center of Respiratory Medicine, Xiangya Hospital, Central South University, Changsha, Hunan China

**Keywords:** COPD, Sleep quality, Symptom change, Exacerbation

## Abstract

**Purpose:**

Study the impact of impaired sleep quality on symptom change and future exacerbation of chronic obstructive pulmonary disease (COPD) patients.

**Methods:**

This was a prospective study. Patients with COPD were recruited into the study and followed up for one year. Pittsburgh sleep quality index (PSQI) was collected at baseline. Symptom change was assessed with Minimum clinically important difference (MCID) in COPD Assessment Test (CAT) at 6-month visit, which is an indicator to assess symptom improvement. Exacerbation was recorded during the one-year visit. PSQI score > 5 was defined as poor sleep quality, whereas PSQI score ≤ 5 was defined as good sleep quality. MCID was defined as attaining a CAT decrease ≥ 2.

**Results:**

A total of 461 patients were enrolled for final analysis. Two hundred twenty-eight (49.4%) patients had poor sleep quality. Overall, 224 (48.6%) patients attained MCID at 6-month visit and the incidence of exacerbation during the one-year visit was 39.3%. Fewer patients with impaired sleep quality achieved MCID than patients with good sleep quality. Good sleepers were significantly more likely to attain MCID (OR: 3.112, *p* < 0.001) than poor sleepers. Fewer poor sleepers in GOLD A and D groups attained MCID with ICS/LABA, and fewer poor sleepers in the GOLD D group attained MCID with ICS/LABA/LAMA than good sleepers. Poor sleep quality was a greater risk factor of future exacerbation in Cox regression analysis. The ROC curves showed that PSQI score had a predictive capacity for future exacerbation. More patients with poor sleep quality experienced future exacerbation in GOLD B and D group with treatment of ICS/LABA/LAMA compared to good sleepers.

**Conclusions:**

COPD patients with impaired sleep quality were less likely to achieve symptom improvement and were at increased risk of future exacerbation compared to patients with good sleep quality. Besides, sleep disturbance may affect the symptom improvement and future exacerbation of patients with different inhaled medication or in different GOLD groups.

**Supplementary Information:**

The online version contains supplementary material available at 10.1186/s12931-023-02405-6.

## Introduction

Chronic obstructive pulmonary disease (COPD) is a chronic respiratory disease with persistent airflow limitation caused by toxic particles or gases [1]. Globally, 174.5 million (2.4%) people suffer from COPD [2], and the prevalence in patients aged 40 years and older in China is 13.7% [3]. COPD is the one of third leading causes of death worldwide [4].

The symptoms of COPD patients include, but are not limited to, dyspnoea, cough, and wheezing, as well as poor sleep quality, depression, and fatigue [5, 6]. Compared with healthy control subjects, poor sleep quality is more frequent in COPD patients. The prevalence of sleep-disordered breathing is reported to be between 50% and 60% in patients with COPD [7, 8]. The most common symptoms include sleep onset insomnia, nighttime awakenings, and sleep deprivation [9, 10]. Factors reported to affect sleep quality in COPD patients include respiratory symptoms [11], depression and anxiety [12], and obstructive sleep apnoea syndrome (OSAS) [13].

The Pittsburgh Sleep Quality Index (PSQI) is a questionnaire mainly used to assess the subjective sleep quality of subjects [14]. A higher PSQI score represents poorer sleep quality. A study demonstrated that PSQI scores of COPD patients were higher compared with individuals without COPD, and sleep quality worsens with disease severity [11, 15]. It has been indicated that patients with a COPD Assessment Test (CAT) ≥ 10 points, or a Modified Medical Research Council Dyspnea Scale (mMRC) ≥ 2 points, had impaired sleep quality [11]. What’s more, at least one daytime COPD symptom, such as dyspnoea or cough, had a negative influence on sleep quality [16–18]. Current studies show that poor sleep quality is one of the important predictors of adverse disease outcomes and mortality [19, 20]. Shorofsky et al. [21] showed that higher PSQI scores were associated with increased risk of future exacerbation and earlier deterioration [21]. Even though bad sleep quality in COPD patients has been shown to be associated with severity of disease and risk of exacerbation, no current study has reported whether poor sleep quality would affect the symptom change in COPD. Therefore, the purpose of this study is to explore the impact of impaired sleep quality on symptoms change and future exacerbation in COPD patients.

## Methods

### Study design and subjects

This was a prospective study. All subjects were obtained from the outpatient COPD database of the Second Xiangya Hospital of Central South University (ChiCTR-POC-17,010,431) from October 2020 to June 2021. According to the Global Initiative for Chronic Obstructive Lung Disease (GOLD) 2020 report, COPD is diagnosed when a ratio of forced expiratory volume in 1 s to forced vital capacity (FEV1/FVC) < 0.70 after inhaling a bronchodilator [22]. The study excluded patients with a history of bronchiectasis, asthma, lung cancer, or pneumonia, or severe heart, liver, or kidney disease.

This study was conducted in accordance with the Declaration of Helsinki and approved by the Ethics Committee of the Second Xiangya Hospital of Central South University. All patients provided written informed consent.

### Data collection

All patients accepted three visits. Clinical characteristics collected at baseline visit included age, sex, education degree, body mass index (BMI), smoking history, biofuel and occupational exposure history, pulmonary function data, CAT, mMRC, Clinical COPD Questionnaire (CCQ), pulmonary function, inhalation therapy drugs (including long-acting muscarinic antagonist (LAMA), inhaled corticosteroids (ICS)/long-acting β2-agonists (LABA), LABA/LAMA, and ICS/LABA/LAMA, exacerbation (moderate-to-severe) in the previous year, PSQI, Hospital Anxiety and Depression Scale (HADS), and Berlin Questionnaire. All patients were followed up for 12 months. Patients were followed up at outpatient centre at 6-month visit, we evaluated the symptom by CAT score, as well as inquired and recorded exacerbations the patients within 6 months. Then, data on exacerbation within latter 6 months were collected at the 12-month follow-up by phone calls.

### Definition and measure

The PSQI is a questionnaire mainly used to assess the subjective sleep quality of subjects and consists of 19 questions. Questions include subjective sleep quality, latency, duration, efficiency, disturbances, use of sleep medications, and daytime dysfunction. Total scores range from 0 to 21. A score > 5 defines poor sleep quality [23], thus the patients were divided into two groups: good sleeper and poor sleeper. A validated three-factor analysis was also used, which is statistically more reliable than a single score [24]. Factor 1, Sleep Efficiency, includes sleep duration and efficiency components (score 0–6). Factor 2, Sleep Quality, includes the perceived sleep quality, sleep latency, and sleep medication use components (score 0–9). Factor 3, Daily Disturbances, includes sleep disturbances (bathroom use, breathing issues, pain) and sleep-related daytime dysfunction (sleepiness, enthusiasm) components (score 0–6).

The HADS is mainly used to assess the degree of anxiety and depression. The total score of both anxiety and depression is 21, and a score > 7 is regarded as the cut-off point for determining anxiety and depression.

The Berlin Questionnaire is a screening scale to assess risk for OSAS. Patients with positive screening and at risk of OSAS were advised to see a specialist outpatient clinic, though how many people used non-invasive positive pressure ventilation wasn’t tracked.

According to the GOLD 2020 report [22], patients were assigned to four categories. Briefly, Group A: 0 to 1 exacerbation per year, no hospitalization, CAT score < 10 or mMRC score of 0 to 1; Group B: 0 to 1 exacerbation per year, no hospitalization, CAT score ≥ 10 or mMRC score ≥ 2; Group C: exacerbation ≥ 2 or hospitalization ≥ 1 per year, CAT score < 10 or mMRC score of 0 to 1; Group D: exacerbation ≥ 2 or hospitalization ≥ 1 per year, CAT score ≥ 10 or mMRC score ≥ 2.

### Assessment for symptom change

CAT score was used to assessed the symptom of COPD. Change in CAT was defined as CAT score changing between baseline and the 6-month visit. The study evaluated the symptoms change with minimum clinically important difference (MCID) during the 6-month follow-up. Minimum clinically important difference was defined as attaining a CAT score decrease ≥ 2 from baseline at the 6-month visit, which is an indicator to assess symptom improvement [25]. The MCID response rate was calculated based on the proportions of individual patients with a ≥ 2 improvement from baseline in CAT score.

### Exacerbations

The study also recorded the incidence of moderate-to-severe exacerbation during the one-year follow-up period. Moderate exacerbation was defined as exacerbation of respiratory symptoms requiring antibiotics and/or oral corticosteroids; severe exacerbation was defined as exacerbation requiring hospitalization or emergency room admission for more than 2 days during the follow-up period. Frequent exacerbation was defined as at least two exacerbations during the follow-up period.

### Statistical analysis

SPSS 26.0 (IBM, Armonk, NY, USA) was used for statistical analysis of the data. Continuous variables were expressed as mean ± standard deviation or median and interquartile range as appropriate. Continuous variables were tested using Student’s t-test; otherwise, non-parametric tests were used. The chi-square test was used for categorical variables. The multivariate stepwise logistic regression analysis was used to analyze the clinical features including sleep quality associated with MCID. A multivariate Cox regression analysis was performed for identifying factors predicting exacerbation during one-year follow up, by including variables that were significant (*p* < 0.05) on univariate analysis. The receiver operating characteristic (ROC) curve was calculated and the area under the curve (AUC) was compared using the Z-test. For all analyses, a *p*-value of < 0.05 was considered statistically significant.

## Results

### Participant characteristics

**Fig. 1:**
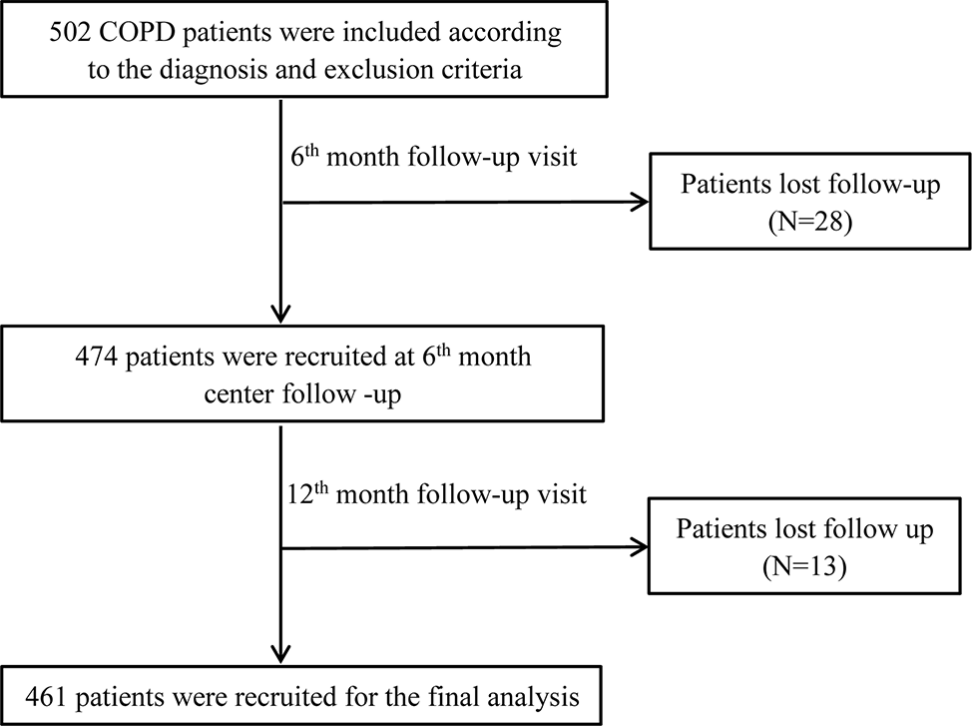
Flow diagram of the inclusion of study **Abbreviations:** COPD, Chronic Obstructive Pulmonary Diseases.

A total of 502 patients with COPD were initially enrolled. At the 6-month visit, 28 patients were excluded from the study due to loss of contact. At the 12-month follow-up, 13 patients dropped out due to loss of contact. Subsequently, 461 patients were recruited for the final analysis (Fig. 1).

As shown in Table 1, the mean age of patients was 63.1 ± 8.2 years and 88.9% of patients were male. The mean CAT score was 12.4 ± 6.8 and the median (IQR) FEV1% was 54.4 (31.2). Most patients were classified as GOLD B (42.3%) and D (34.7%). The mean PSQI score was 5.8 ± 3.4, 228 (49.4%) patients had poor quality sleep. The proportion of patients at high risk for OSAS is 20.1%. Thirty-one (6.8%) patients had anxiety and 12 (2.4%) had depressive tendencies.

**Table 1:** The distribution of baseline demographic and clinical characteristics according to sleep quality

Variables	Total	Bad sleeper	Good sleeper	*P*- value
Number of patients, n (%)	461(100.0)	228(49.5)	233(50.5)	
Age (years)^a^	63.1 ± 8.2	64.3 ± 8.5	62.1 ± 9.0	0.016
Sex^b^				0.287
Male	410(88.9)	199(87.3)	211(90.6)	
Female	52(11.1)	29(12.7)	22(9.4)	
Education^b^				0.116
Primary school	158(34.3)	84(36.8)	74(31.8)	
Junior high school	189(41.0)	97(42.5)	92(39.5)	
High school	77 (16.7)	35(15.4)	42(18.0)	
University	37(8.0)	12 (5.3)	25 (10.7)	
BMI (kg/m^2^)^a^	22.6 ± 3.1	22.3 ± 3.2	22.9 ± 3.4	0.096
Marry status^b^				0.354
married	351 (95.4)	219 (96.1)	222(95.2)	
unmarried	17(4.6)	9(3.9)	11 (4.8)	
Smoking state^b^				0.614
Current smoker	178(38.8)	84(36.9)	95(40.7)	
Ex-smoker	210(45.6)	107(46.9)	103(44.2)	
Non-smoker	72(16.6)	37(16.2)	35(16.0)	
Biofuel exposure^b^				0.292
Yes	169(36.7)	90(39.3)	79(34.1)	
No	292(63.3)	138(60.7))	154 (65.9)	
Occupational^b^				0.608
Yes	182(39.4)	90(39.3)	79(39.5)	
No	279(60.6)	138 (60.7))	154 (60.5)	
CAT^a^	12.4 ± 6.8	14.6 ± 6.3	9.8 ± 6.0	<0.001
mMRC^b^	2 (1)	2(2)	2(1)	<0.001
0–1	179(38.8)	71(31.1)	108(46.4)	0.001
2–4	282(61.2)	157(68.9)	125(53.6)	
CCQ^a^	18.4 ± 8.2	20.3 ± 7.7	17.7 ± 7.4	<0.001
FEV1(L)^c^	1.4(0.90)	1.33(0.73)	1.49(0.99)	0.031
FEV1 (% predicted)^c^	54.40(31.2)	52.5(28.7)	56.0(31.7)	0.178
FEV1/FVC^c^	50.9(19.9)	47.1(19.7)	51.3(23.9)	0.030
Exacerbations in the past year^c^	0(1)	0(1)	0(1)	0.258
Exacerbations in the past year^b^				0.404
0	230(49.9)	109(47.8)	121 (51.9)	
1	122(26.4)	59 (25.9)	63 (27.0)	
≥ 2	109(23.7)	60 (26.3)	49(21.1)	
GOLD group^b^				<0.001
A	81(17.6)	23(10.1)	58(24.9)	
B	195(42.3)	110(48.2)	85(36.5)	
C	25(5.4)	5(2.2)	20(8.6)	
D	160(34.7)	90(39.5)	70(30.0)	
Treatment^b^				0.012
LAMA	91(19.7)	33(14.5)	58(24.9)	
LABA + ICS	62(13.4)	31(13.6)	31(13.3)	
LABA + LAMA	61(13.2)	37(16.2)	24(10.3)	
LABA + LAMA + ICS	217(47.1)	106(46.5)	111(47.6)	
Others	30(6.5)	21(9.2)	9(3.9)	
PSQI score^a^	5.8 ± 3.4	8.5 ± 2.7	3.2 ± 1.4	<0.001
Sleep efficiency^a^	1.6 ± 1.1	2.3 ± 0.9	0.8 ± 0.6	<0.001
Sleep quality^a^	2 ± 1.4	3.2 ± 1.2	1.3 ± 0.6	<0.001
Daily disturbance^a^	2.0 ± 1.0	2.9 ± 1.1	1.1 ± 0.7	<0.001
Risk of OSAS^b^				0.892
positive	93 (20.1)	46 (20.2)	47 (20.0)	0.890
negative	368 (79.9)	182 (79.8)	186 (80.0)	
Anxiety^b^	31 (6.8)	22 (9.8)	9 (3.8)	0.030
Depresion^b^	11 (2.4)	8 (3.3)	3 (1.1)	0.053

^a^Mean ± SD; ^b^Counts with percentage are indicated; ^c^Median (IQR)

**Abbreviations:** BMI, Body Mass Index; COPD, Chronic Obstructive Pulmonary Diseas; CAT, COPD Assessment Test; CCQ, Clinical COPD Questionnaire; FEV1, Forced Expiratory Volume in one second; FVC, Forced Vital Capacity; GOLD, Global Initiative for Chronic Obstructive Lung Disease. ICS, inhaled corticosteroids; IQR, interquartile range; LABA, long-acting β-2-agonist; LAMA, long-acting muscarinic antagonist; mMRC, modified medical research council dyspnea scale; OSAS, obstructive sleep apnea syndrome ; PSQI, Pittsburgh sleep quality index.

Compared with good sleepers, poor sleepers were older. Poor sleepers had lower absolute FEV1 value and lower FEV1/FVC, but higher baseline CAT and CCQ scores. In addition, more poor sleepers had an mMRC > 1. Furthermore, poor sleepers had a higher proportion of patients in GOLD B and D groups. There was no difference in risk of OSAS between the two groups. However, more poor sleepers had anxiety (Table 1). There was no difference in baseline clinical features in patients with risk of OSAS and without risk of OSAS (Supplement Table 1).

### Sleep quality and symptom change

**Fig. 2:**
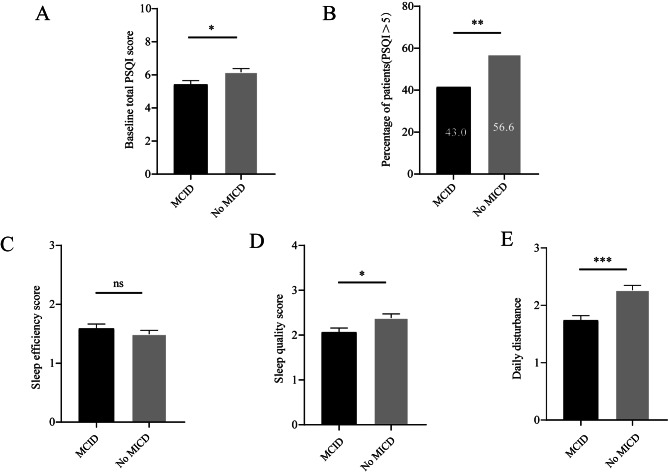
**Baseline PSQI score in those with MCID and without MCID.** **Note**: (A) Baseline total PSQI score in COPD patients with MICD and without MICD; (B) Percentage of patients with PSQI > 5 in COPD patients with MICD and without MICD; (C) Sleep quality score in COPD patients with MICD and without MICD; (D) Sleep quality score in COPD patients with MICD and without MICD; (E) Daily disturbance score in COPD patients with MICD and without MICD; ns indicates *P*-values > 0.05;*indicates *P*-values < 0.05, ** indicates *P*-values < 0.01, *** indicates *P*-values < 0.001 **Abbreviations:** MCID, minimum clinically important difference; PSQI, Pittsburgh sleep quality index.

As shown in Table 2, the median change in CAT of all study population was1(8), 224 (48.6%) patients attained MCID in CAT at 6 months. Change in CAT between poor sleepers and good sleepers was different [0(9) vs. 2(6), *p* = 0.021]. Compared with patients with good sleep quality, fewer patients with poor sleep quality obtained MCID [131 (56.2%) vs. 93 (40.8%), *p* = 0.001]. In addition, the mean total PSQI score in patients without MCID was higher than patients with MCID (6.9 ± 3.3 vs. 4.5 ± 2.4, *p* = 0.019), as well as the mean Sleep Quality score (2.4 ± 1.4 vs. 2.0 ± 1.3, *p* = 0.013) and Daily Disturbance score [2.3 ± 1.3 vs. 1.7 ± 1.2, *p* < 0.001] (Fig. 2). Furthermore, there was a greater proportion of PSQI > 5 in patients without MCID than patients with MCID (Fig. 2). After adjusting for sex, age, biofuel exposure, CAT score, mMRC, CCQ score, GOLD group, inhalation drug, sleep quality, risk of OSAS, and anxiety and depression, the multivariate logistic regression showed that a PSQI ≤ 5 (OR: 3.112, 95% CI: 1.873–4.711, *p* < 0.001), higher baseline CAT score (OR: 1.372, 95% CI: 1.274–1.477, *p* < 0.001), and patients with biofuel exposure (OR: 1.102, 95% CI: 1.009–1.214, *p* = 0.036) were more likely to attain MCID at the 6-month visit (Table 3).

**Table 2:** MCID response rate and exacerbation of patient during the one-year visit according to sleep quality

Variables	Total N = 461	Bad sleeper N = 228	Good sleeper N = 233	*P*-value
CAT at 6th month^a^	10.7 ± 6.1	13.6 ± 5.6	7.9 ± 4.7	<0.001
Change in CAT^c^	1(8)	0(9)	2(6)	0.021
MCID of CAT ^b^				
Yes	224(48.6)	93(40.8)	131(56.2)	0.001
No	237(51.4)	135(59.2)	102(43.6)	
Exacerbations in the one year^c^	0(1)	0(1)	0(0)	<0.001
Exacerbation in the one year ^b^				<0.001
Yes	181(39.3)	108(47.4)	73(31.3)	
No	280(60.7)	120(52.6)	160(68.7)	
Severe exacerbation in the one year ^b^				0.017
Yes	117(25.4)	69(30.3)	48(20.6)	
No	344(74.6)	159(69.7)	185(79.4)	
Frequent exacerbation in the one year ^b^				0.008
Yes	65(14.1)	42(18.4)	23(9.9)	
No	396(85.9)	186(81.6)	210(90.1)	

^a^Mean ± SD; ^b^Counts with percentage are indicated; ^c^Median (IQR)

**Abbreviations:** CAT, COPD Assessment Test; MCID, minimum clinically important difference.

**Table 3:** Univariate and Multivariate logistic regression analysis of factors associated with MCID in CAT.

Variable	Univariate			Multivariate		
Factors	OR	95%CI	*P* -value	OR	95%CI	*P* -value
Age (years)	1.013	0.991–1.036	0.245			
Sex (Male versus Female)	0.890	0.502–1.607	0.717			
BMI (kg/m^2^)	0.958	0.895–1.025	0.216			
Smoking state						
Current smoker	Reference					
Ex-smoker	1.386	0.774–2.477	0.271			
Non-smoker	0.995	0.582–1.703	0.986			
Biofuel exposure, (Yes versus No)	1.364	1.126–1.579	0.000	1.102	1.009–1.214	0.036
Occupational exposure (Yes versus No)	1.055	0.717–1.522)	0.786			
CAT score	1.179	1.136–1.225	<0.001	1.372	1.274–1.477	<0.001
mMRC (Median, IQR)						
0–1	Reference					
2–4	1.021	1.002–1.075	0.008			
CCQ score	1.077	1.049–1.106	<0.001			
GOLD group						
A	Reference					
B	2.413	1.616–2.907	0.000			
C	1.043	0.583–1.965	0.934			
D	1.921	1.423–2.214	0.001			
Exacerbations in the past year, (Yes versus No)	1.321	0.914–1.908	0.139			
Treatment						
LAMA	Reference					
LABA + ICS	1.011	0.521–1.961	0.975			
LABA + LAMA	1.988	1.216–3.496	0.011			
LABA + LAMA + ICS	1.894	1.148–3.125	0.012			
PSQI ≤ 5	2.364	1.388–3.268	0.001	3.112	1.873–4.711	<0.001
Risk of OSAS	1.002	0.789–1.314	0.852			
Anxiety	0.957	0.568–1.127	0.431			
Depresion	1.018	0.498–1.637	0.612			

**Notes:** Age, sex, Biofuel exposure, CAT score ,mMRC score, CCQ score, GOLD group, OSAS, anxiety, depression, sleep quality and inhalation drug were included as the variables in the multivariate logistic regression model.

**Abbreviations:** BMI, Body Mass Index; COPD, Chronic Obstructive Pulmonary Disease;GOLD, Global Initiative for Chronic Obstructive Lung Disease. ICS, inhaled corticosteroids; IQR, interquartile range; LABA, long-acting β-2-agonist; LAMA, long-acting muscarinic antagonist; mMRC, modified medical research council ; dyspnea scale; MCID, minimum clinically important difference; OSAS,obstructive sleep apnea syndrome ; PSQI, Pittsburgh sleep quality index.

### Symptom improvement of different GOLD groups or different inhalation therapies in COPD patients with different sleep quality

**Fig. 3:**
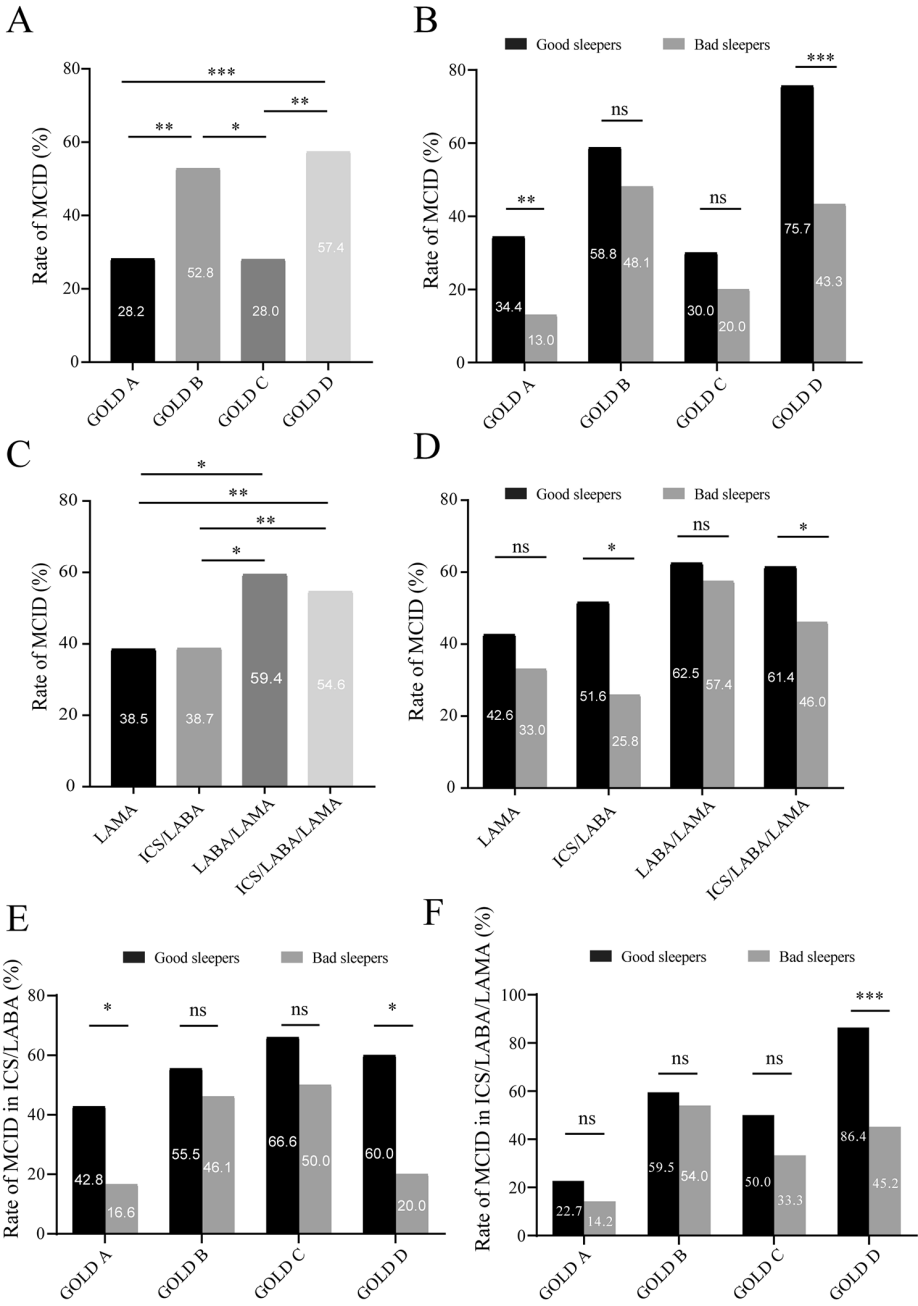
**Comparison of the symptoms improvement between different GOLD group or different main inhalation therapy in COPD patients with different sleep quality** MCID response rate of different GOLD group in COPD patients. (B) MCID response rate of different GOLD group in COPD patients with different sleep quality. (C) MCID response rate of different inhaled medication in COPD patients. (D) MCID response rate of different inhaled medication in COPD patients with different sleep quality. (E) MCID response rate of different GOLD group in treatment of ICS/LABA. (F) MCID response rate of different GOLD group in treatment of ICS/LABA/LAMA. ns indicates *P*-values > 0.05, *indicates *P*-values < 0.05, **indicates *P*-values < 0.01,*** indicates *P*-values < 0.001 **Abbreviations:** GOLD, Global Initiative for Chronic Obstructive Lung Disease; ICS, inhaled corticosteroids; IQR, interquartile range; LABA, long-acting β-2-agonist; LAMA, long-acting muscarinic antagonist; MCID, minimum clinically important difference.

Of all patients, COPD patients in GOLD B and D groups attained a greater proportion of MCID than COPD patients in GOLD A and C groups. The study compared response rates of MCID of different GOLD groups due to sleep quality. Fewer patients with sleep disturbance attained MCID in GOLD A (*p* = 0.008) and GOLD D (*p* < 0.001) groups compared with good sleepers (Fig. 3A and B).

In all participants, COPD patients with LAMA/LABA (38/64, 59.3%) or ICS/LABA/LAMA (116/214, 54.6%) were more likely to get symptom improvement than LAMA (35/91, 38.4%) or ICS/LABA (24/62, 38.7%). Whereas sleep quality—good or poor—had no difference in MCID response rate of LAMA and LABA/LAMA. However, fewer patients with impaired sleep quality achieved MCID with ICS/LABA (*p* = 0.030) and ICS/LABA/LAMA (*p* = 0.024) compared with good sleepers (Fig. 3C and D).

The study further analysed MCID response rates of different GOLD groups with ICS/LABA and ICS/LABA/LAMA. Fewer patients with impaired sleep quality in GOLD A (*p* = 0.031) and D (*p* = 0.042) groups attained MCID with ICS/LABA therapy, and fewer poor sleepers in GOLD D (*p* < 0.001) group obtained MCID with ICS/LABA/LAMA therapy compared to good sleepers (Fig. 3E-F).

### Sleep quality and risk of future exacerbation

Of 461 patients, the median (IQR) number of exacerbations during the one-year visit was 0 (1); 39.3% of patients had at least one moderate-to-severe exacerbation, 25.4% of patients experienced severe exacerbation, and 14.1% of patients experienced frequent exacerbation. In addition, the median (IQR) exacerbation during the one-year visit in poor sleepers and good sleepers were (0, 1) and (0, 0), respectively (*p* < 0.001) (Table 2). More patients with sleep disturbance had higher incidence of moderate-to-severe exacerbation (*p* < 0.001), severe exacerbation (*p* = 0.017), and frequent exacerbation (*p* = 0.008) than good sleepers (Table 2). Patients with moderate-to-severe exacerbation and severe exacerbation during the 12-month visit had higher total PSQI scores, Sleep Efficiency scores, Sleep Quality scores, and Daily Disturbance scores, and greater proportions of PSQI > 5. Patients with frequent exacerbation had higher total PSQI scores, Sleep Efficiency scores, and Daily Disturbance scores, and greater proportions of PSQI > 5, but not higher Sleep Quality scores (Supplement Fig. 1).

**Fig. 4:**
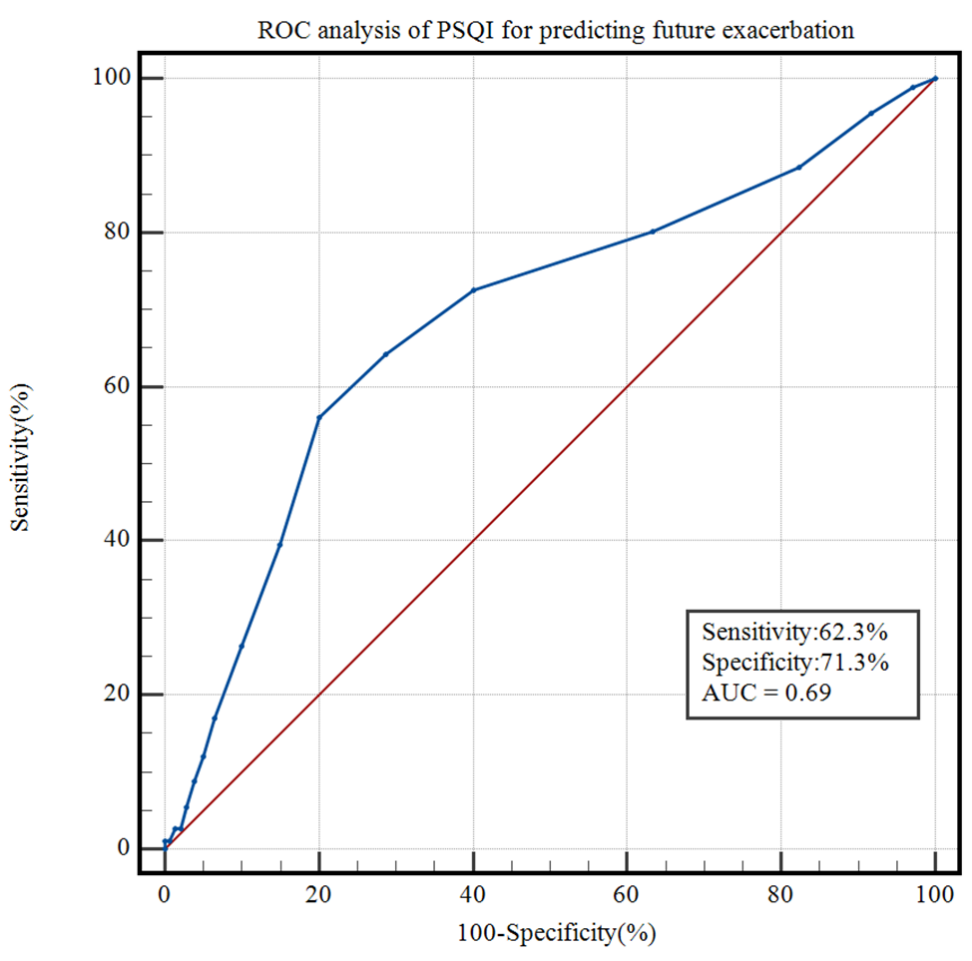
**ROC curve of sleep quality for predicting future exacerbation** AUC of PSQI score for predicting future exacerbation is 0.69(95% CI: 0.64–0.73, *P*<0.001), the sensitivity and the specificity is 62.3% and 71.3% **Abbreviations:** AUC, Area under of ROC curve; PSQI, Pittsburgh sleep quality index; ROC, Receiver operating characteristic.

After adjusting for age, sex, CAT score, mMRC score, CCQ score, GOLD group, exacerbation in the past year, inhalation drug, risk of OSAS, and anxiety and depression, results of Cox analysis revealed that a PSQI > 5 was a great risk factor of moderate-to-severe exacerbation, frequent exacerbation, and severe exacerbation during one-year follow up. The global PSQI score was a significant risk factor of moderate-to-severe exacerbation and frequent exacerbation, but not severe exacerbation during the one-year follow-up period (Table 4). The ROC curve exhibited that future exacerbation of COPD patients was predicted by baseline PSQI score (AUC: 0.69, 95% CI: 0.64–0.73, *p* < 0.001) (Fig. 4).

**Table 4:** Risk of sleep quality for future exacerbation during one- year follow-up using Cox regression

	Moderate to severe exacerbation		Frequent exacerbation		Severe exacerbation	
	HR (95CI%)	*P-* value	HR (95CI%)	*P-* value	HR (95CI%)	*P-* value
PSQI>5*	1.692(1.213–2.357)	0.002	1.766(1.115–2.797)	0.015	1.124(1.018–1.301)	0.031
PSQI^#^	1.070(1.030–1.111)	<0.001	1.080(1.020–1.142)	0.008	1.010(0.983–1.026)	0.102

**Notes:** Multivariate cox analysis of model 1(*) and model2 (#) were adjusted for age, sex, CAT score, mMRC score, CCQ score, GOLD group, exacerbation in the past year, inhalation drug, risk of OSAS, anxiety and depression.

**Abbreviations:** CAT, COPD Assessment Test; CCQ, Clinical COPD Questionnaire; GOLD, Global Initiative for Chronic Obstructive Lung Disease; HR, hazard rate? mMRC, modified medical research council dyspnea scale; MCID, minimum clinically important difference; OSAS, obstructive sleep apnea syndrome ; PSQI, Pittsburgh sleep quality index.

### Occurrence of future exacerbation between different GOLD groups or different inhalation therapies in COPD patients with different sleep quality

**Fig. 5:**
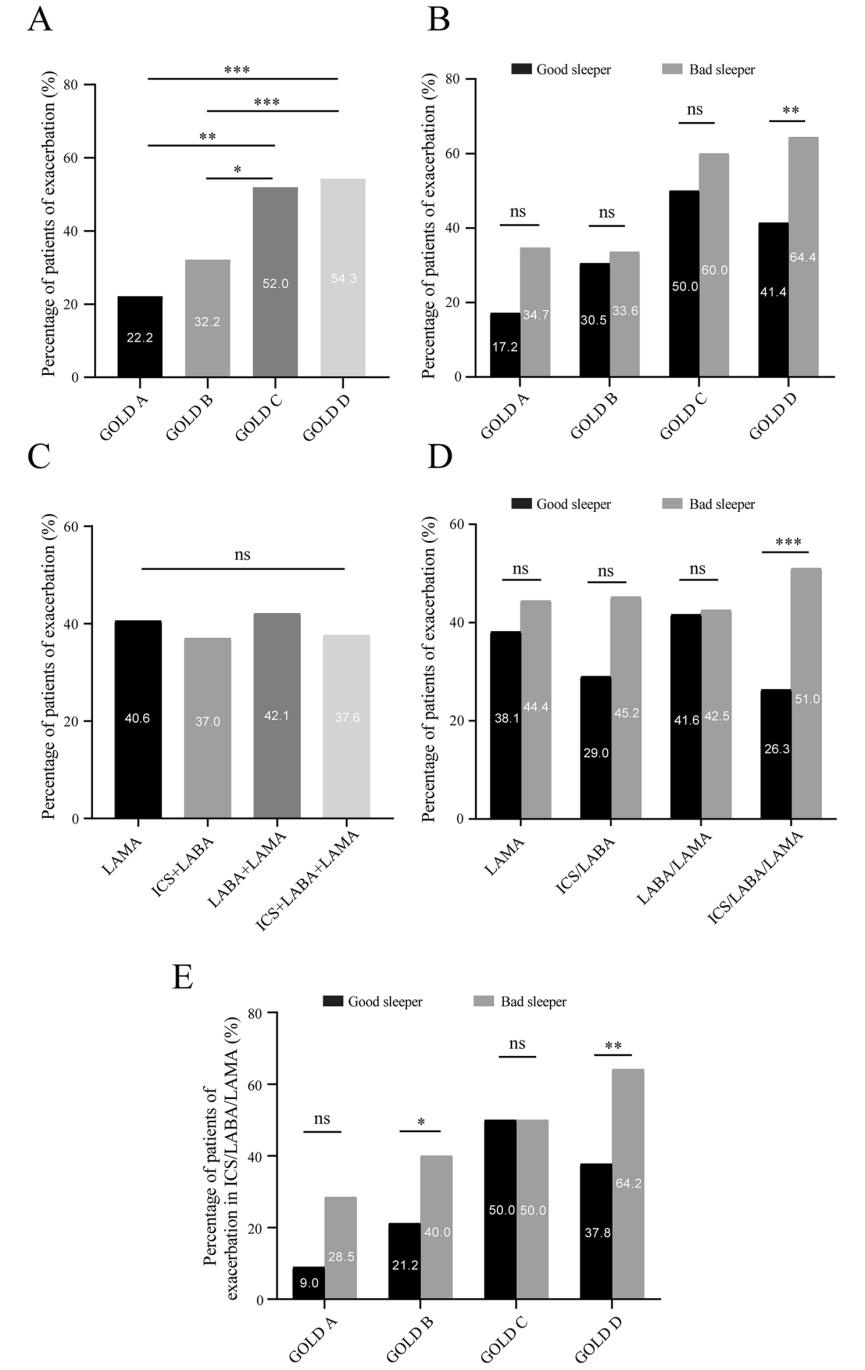
**Comparison of the incidence of exacerbation between different GOLD group or different main inhalation therapy in COPD patients with different sleep quality** Incidence of future exacerbation of different GOLD group in COPD patients. (B) Incidence of future exacerbation of different GOLD group in COPD patients with different sleep quality. (C) Incidence of future exacerbation of different inhaled medication in COPD patients. (D) Incidence of future exacerbation of different inhaled medication in COPD patients with different sleep quality. (E) Incidence of future exacerbation of different GOLD different in treatment of ICS/LABA/LAMA. ns indicates P-values > 0.05;*indicates *P*-values < 0.05, **indicates *P*-values < 0.01,*** indicates *P*-values < 0.001

Patients in GOLD C and GOLD D groups experienced higher incidence of exacerbation during the one-year visit than GOLD A and B groups. There was no significant difference in incidence of exacerbation in GOLD A, B, and C groups between patients with different sleep quality. However, more patients with impaired sleep quality experienced future exacerbation in GOLD D group than good sleepers (Fig. 5A and B).

Furthermore, there was no difference in incidence of future exacerbation in patients with different inhaled medication treatment. More poor sleepers experienced future exacerbation in the one-year follow up with ICS/LABA/LAMA (*p* < 0.001), whereas there was no difference in incidence of exacerbation of LAMA, ICS/LABA, and LABA/LAMA between patients with poor sleep quality and good sleep quality (Fig. 5C and D).

The study further analysed the incidence of future exacerbation of different GOLD groups with ICS/LABA/LAMA. More poor sleepers experienced future exacerbation in GOLD B (*p* = 0.046) and D (*p* = 0.009) groups with ICS/LABA/LAMA than good sleepers (Fig. 5E).

## Discussion

The study indicated that fewer patients with poor sleep quality attained symptom improvement than good sleepers. In our study, we used MCID (CAT improved ≥ 2) to evaluate the change in symptom. MCID was the CAT responsive to short-term changes in COPD patients and is an indicator to assess symptom improvement [25]. The mean total PSQI score, Sleep Quality score, and Daily Disturbance score, and proportions of PSQI > 5 were higher in patients without MCID compared with patients with MCID. This was the first study to find the correlation between sleep quality of COPD patients and change in CAT at 6 months. In addition, a PSQI ≤ 5 was a significant factor related with related with clinically significant reduction in CAT score in multivariate logistic regression. Previous studies also demonstrated that patients with poor sleep quality may experience worse quality of life and disease outcomes, including exacerbation and mortality [20]. However, short-term clinically important deterioration (including change of CAT score) was associated with long-term exacerbation and mortality risk [26, 27]. Therefore, it may be consistent with the result of this study, in which COPD patients with good sleep quality are more likely to obtain MCID in CAT and patients with sleep disturbance are less likely to obtain MCID in the short term. However, previous studies have not specifically investigated whether poor sleep quality affected improvement in symptoms. Multivariate logistic regression also revealed that a higher baseline CAT score was one of the independent correlation factors for MCID. Although patients with sleep disturbances had higher baseline CAT scores, they were less likely to attain improvement in CAT, with smaller proportions of MCID in the short term; therefore, it will be an important subtype to focus on.

Results of this study also indicated that poor sleepers had higher baseline CAT and CCQ scores and more proportions with an mMRC > 1 than good sleepers. These results are similar with the reported study that patients with CAT ≥ 10 points, or mMRC ≥ 2 points, in GOLD B and D groups were more likely to have impaired sleep quality [11].

This study also presented that the mean PSQI score was 5.8 ± 3.4 and 49.5% of COPD patients with a PSQI > 5 had impaired sleep quality. This result is consistent with previous studies in which the mean PSQI score was 6.4 ± 3.9 and 51.0% of COPD patients had impaired sleep quality [6]. The results also demonstrated that 48.6% of COPD patients attained MCID at 6 months. A recent study also indicated that 51% of patients treated with inhalation therapy achieved a MCID of any two measures such as CAT, St. George’s Respiratory Questionnaire (SGRQ), Self-Administered Computerized-Transition Dyspnea Index (SAC-TDI), and Evaluating Respiratory Symptoms (E-RS) at the 24th week [28].

Furthermore, the study elucidated that patients with future exacerbation had higher total PSQI scores and greater proportions of PSQI > 5 than patients without future exacerbation. It was mostly in line with the published study in which individuals with exacerbation during the 18-month follow-up period had a higher baseline global PSQI score and were also more likely to be poor sleepers [21]. Cox regression analysis demonstrated that poor sleep quality was a greater risk factor of moderate-to-severe exacerbation, frequent exacerbation, and severe exacerbation during one-year follow up. The global PSQI score was a significant risk factor of moderate- to-severe exacerbation and frequent exacerbation, but not severe exacerbation. A reported study using the Negative Binomial Regression Model also revealed that a higher baseline global PSQI score and a PSQI score > 5 were associated with a greater risk of symptom-based or event-based exacerbation [21]. The difference is that the result in our study analysed the relationship between sleep quality and risk of moderate-to-severe exacerbation, severe exacerbation, and frequent exacerbation in Cox analysis, while the previous study focused on correlation between sleep quality and symptom-based exacerbation and event-based exacerbation in the Negative Binomial Model. Besides, our study is the first study to illustrate that sleep quality has capacity for predicting future exacerbation using ROC curve, with an AUC of 0.69.

There are some limitations of this study. Firstly, recent study revealed that OSAS may have impact on sleep quality. This study did not exclude COPD patients with OSAS, but our study assessed the risk of OSAS in patients and adjusted the risk of OSAS in multivariate analysis. Secondly, it has not been definitively established that a PSQI > 5 is the best cut-off for poor sleepers in a COPD population. Although a PSQI > 5 was originally developed for healthy individuals, many studies have already used the cut-off value in COPD patients. Future work is needed as we come to understand more about the relationship between sleep and COPD.

## Conclusions

COPD patients with impaired sleep quality were less likely to obtain symptom improvement and were at increased risk of future exacerbation than patients with good sleep quality. Impaired sleep quality may also affect the symptom improvement and future exacerbation of patients with different inhaled medication or in different GOLD groups. This study may provide a clinical basis of relation between sleep quality and choice of inhalation medication.

## Electronic supplementary material

Below is the link to the electronic supplementary material.

Supplementary Material 1

## Data Availability

All publications discussed in the manuscript are available from the corresponding author on request.

## Abbreviations

BMI: Body Mass Index
COPD: Chronic Obstructive Pulmonary Disease;
CAT: COPD Assessment Test
FEV1: Forced Expiratory Volume in one second
FVC: Forced Vital Capacity
GOLD: Global Initiative for Chronic Obstructive Lung Disease
HADS: Hospital Anxiety and Depression Scale
ICS: inhaled corticosteroids
IQR: interquartile range
LABA: long-actingβ-2-agonist
LAMA: long-acting muscarinic antagonist
MCID: minimal clinically important difference
mMRC: modified Medical Research Council
OSAS: obstructive sleep apnea syndrome
PSQI: Pittsburgh sleep quality index
ROC: The receiver operating characteristic
SGRQ: St George’s Respiratory Questionnaire
