# Supplementary material for: The impact of impaired sleep quality on symptom change and future exacerbation of chronic obstructive pulmonary disease
Source: Respir Res. 2023 Mar 30;24:98. doi: 10.1186/s12931-023-02405-6 (PMC10064786; doi:10.1186/s12931-023-02405-6)
Supplement: Supplementary file 1 — Supplementary Material 1 [file 12931_2023_2405_MOESM1_ESM.doc]

**Supplement Table 1: Clinical characteristic in COPD patients with and without risk of OSAS**

| **Variables** | **Patients with OSAS**  **N=93** | **Patients without OSAS**  **N=368** | **P-**  **value** |
| --- | --- | --- | --- |
| Age (years)^a^ | 63.0± 8.7 | 62.5 ± 8.8 | 0.629 |
| Male^b^ | 82(88.2) | 328(80.0) | 0.249 |
| BMI (kg/m^2^)^a^ | 22.9 ±3.5 | 22.0± 3.0 | 0.076 |
| Marry status^b^ |  |  | 0.554 |
| married | 89 (95.7) | 351(95.4) |  |
| unmarried | 4(4.3 | 15 (4.6) |  |
| Current smoker^b^ | 34(36.6) | 144(39.1) | 0.409 |
| Biofuel exposure^b^ | 30(32.3) | 139(37.8) | 0.295 |
| Occupational exposure^b^ | 38(40.8) | 144(39.1) | 0.888 |
| CAT^a^ | 13.6±7.5 | 12.4± 6.7 | 0.112 |
| mMRC^c^ | 2(1) | 2(1) | 0.467 |
| CCQ^a^ | 21.6 ± 7.7 | 19.8 ± 8.2 | 0.280 |
| FEV1(L)^c^ | 1.37(0.71) | 1.43(0.85) | 0.467 |
| FEV1(% predicted)^c^ | 55.8(28.7) | 54.0(24.2) | 0.375 |
| FEV1/FVC^c^ | 53.0(23.1) | 48.1(21.5) | 0.038 |
| PSQI score^a^ | 6.4±4.0 | 5.9±3.5 | 0.257 |
| Anxiety^b^ | 6 (6.5) | 25 (6.7) | 0.497 |
| Depresion^b^ | 3 (3.2) | 8 (2.2) | 0.326 |
| Exacerbations  in the past year^c^ | 0(1) | 0(1) | 0.627 |
| Exacerbations  in the past year^b^ |  |  | 0.627 |
| 0 | 44(47.3) | 186 (50.5) |  |
| ≥1 | 49 (52.7) | 182 (49.5) |  |
| Treatment^b^ |  |  | 0.408 |
| LAMA | 18(19.3) | 73(19.8) |  |
| LABA+ICS | 12(12.9) | 50(13.6) |  |
| LABA+LAMA | 15(16.1) | 46(12.5) |  |
| LABA+LAMA+ICS | 43(46.2) | 174(47.3) |  |
| Others | 5(5.5) | 25(5.4) |  |

^a^Mean ± SD; ^b^Counts with percentage are indicated; ^c^Median (IQR)

**Abbreviations:** BMI, Body Mass Index; COPD, Chronic Obstructive Pulmonary Diseas; CAT, COPD Assessment Test; CCQ, Clinical COPD Questionnaire; FEV1, Forced Expiratory Volume in one second; FVC, Forced Vital Capacity; GOLD, Global Initiative for Chronic Obstructive Lung Disease. ICS, inhaled corticosteroids; IQR, interquartile range; LABA, long-acting β-2-agonist; LAMA, long-acting muscarinic antagonist; mMRC, modified medical research council dyspnea scale;

PSQI, Pittsburgh sleep quality index.

**
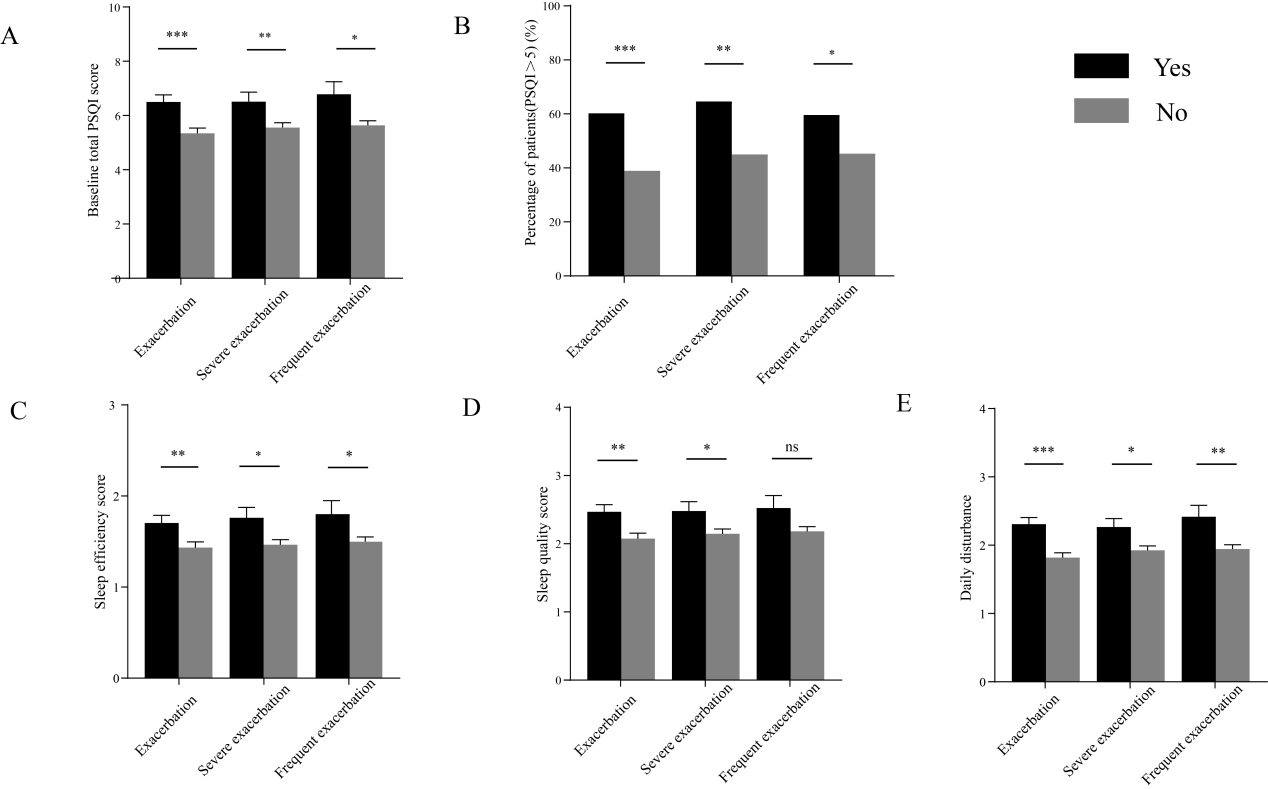
**

**Supplement Figure 1. Baseline PSQI score in those with and without exacerbation during the one-year Follow-up Period**

1. Baseline total PSQI score in patients with and without at least one moderate to severe exacerbation group, different future severe exacerbation group and different future frequent exacerbation group. (B) Percentage of patients with PSQI＞5 in patients with and without at least one moderate to severe exacerbation group, different future severe exacerbation group and different future frequent exacerbation group. (C,D,E) sleep efficiency, sleep quality and daily disturbance in in patients with and without future at least one moderate to severe exacerbation group, severe exacerbation group and future frequent exacerbation group. *indicates p-values <0.05, ** indicates p-values <0.01, *** indicates p-values <0.001.

**Abbreviations:**  PSQI, Pittsburgh sleep quality index.
